# Evaluation of swallowing in children with higher grades glottic web

**DOI:** 10.1007/s00405-023-08127-1

**Published:** 2023-07-27

**Authors:** Ahmed El-Sobki, Reham AE Ibrahim, Saad Elzayat, Mohamed E. El-Deeb, Bassem Ashraf, Menna Ibrahim Hashish, Mahmoud Elsaid Ibrahim Alsobky, Abeer Salamah

**Affiliations:** 1 Otorhinolaryngology Department, Faculty of Medicine, Mansoura University, Mansoura, Egypt; 2 Phoniatrics Department, Faculty of Medicine, Assiut University, Assiut, Egypt; 3 Otorhinolaryngology Department, Faculty of Medicine, Kafrelsheikh University, Kafrelsheikh, Egypt; 4 Pediatrics Department, Faculty of Medicine, Mansoura University, Mansoura, Egypt; 5 Otorhinolaryngology Department, Faculty of Medicine-Damietta, Alazhar University, Damietta, Egypt; 6 Pediatrics Department, Faculty of Medicine, Kafrelsheikh University, Kafrelsheikh, Egypt

**Keywords:** Swallowing disorders, Aspiration, Laryngeal web, Surgery, Children

## Abstract

**Purpose:**

to evaluate the swallowing function in children with higher grades of glottic web and to detect the impact of surgical division of the glottic web on the swallowing parameters. We also performed a voice analysis as a secondary objective in this study.

**Methods:**

This prospective case series study included 12 children with higher grades of the glottic web; grades 3 and 4. Evaluation of the swallowing function was done by clinical swallowing evaluation including symptoms and signs of swallowing dysfunction during feeding, such as vomiting, coughing, choking, or cyanosis, and bedside swallowing assessment using the 3-oz water swallow test. Instrumental evaluation of swallowing function was performed using flexible endoscopic evaluation of swallowing (FEES). The evaluation was performed both preoperatively and postoperatively.

**Results:**

The number of children suffering from swallowing difficulties significantly increased during the postoperative evaluation where 6 (50%) children demonstrated choking during feeding after the surgical division of the web in comparison to only 3 (25%) preoperatively. Also, coughing and choking during the 3-oz water swallow test significantly increased following the division of the web with *P* < 0.001.

**Conclusion:**

Swallowing assessment is mandatory as children with higher grades of the glottic web, requiring reconstructive surgeries, are at risk of swallowing deficit which can be aggravated postoperatively. With improvement in the airway and surgery-specific outcomes, swallowing function is an important secondary outcome that has a significant impact on the lives of these kids and their families.

## Introduction

Congenital webs make up less than 5% of all congenital laryngeal anomalies. They are caused by failure of recanalization of the larynx in the tenth week of embryogenesis [1].

Most congenital webs are considered a type of laryngeal atresia instead of a real web, and they manifest as a thick fibrotic web with subglottic stenosis. The main symptoms include dysphonia, hoarseness, biphasic stridor, recurrent croup, or pneumonia. In extreme cases, apnea, cyanosis, and failure to thrive are prevalent [2].

An initial evaluation should be performed by flexible laryngoscopy to rule out other pathologies such as laryngomalacia and other laryngeal anomalies. Also, the rigid bronchoscopy is performed for definitive evaluation, and to assess the severity of the web and its subglottic extension [3].

Cohen [4] classified the glottic web according to the degree of glottic narrowing into four types (1–4). The more severe webs extend into the subglottic region causing subglottic stenosis. A type 3 web has glottic involvement of 50–75%, while a type 4 web entails 75–90% of the glottis. Both types are associated with subglottic stenosis (SGS).

The anterior glottic web is a difficult clinical entity to treat and the main difficulty has been web reformation after surgery. The airway obstruction severity and the web extension determine management strategies [5].

High-grade glottic webs typically cause airway symptoms and usually necessitate tracheostomy early in life. The endoscopic approach is not enough in these cases due to the associated cartilaginous subglottic stenosis which necessitates open surgery [6].

The airway obstruction and associated inspiratory stridor usually increase with the respiratory effort encountered during feeding which creates a sort of imbalance or incoordination between breathing and swallowing and may lead to abnormalities in swallowing function up to aspiration that may pass unnoticed in some cases.

Therefore, this study was conducted to evaluate the swallowing function in children with higher grades of glottic web and to detect the impact of surgical division of the glottic web on the swallowing parameters. We also performed a voice analysis as a secondary objective in this study.

## Patients and methods

This prospective case series study included 12 children with higher grades of the glottic web; grades 3 and 4. It was held in the Otolaryngology Departments of Mansoura, Kafrelsheikh, and Assiut University hospitals over 3 years period from August 2019 to July 2022. This research was approved by the university ethical committee and written approved consent was obtained from the parents of included patients (MKSU 50-2-9).

### Examination

Examination under anesthesia was performed, slim 30-degree rigid endoscopy was performed, while the baby was spontaneously breathing to evaluate the thickness of the web and associated subglottic stenosis. Gentle manipulation was mandatory not to pass to undesirable airway emergencies. All cases were always evaluated by a pediatrician and genetic therapist. Echocardiography was performed in all cases to assess associated cardiac anomalies. A screening fluorescence in-situ hybridization (FISH) test for a deletion at chromosome 22q11.2 locus was performed on the cases. Three cases were referred to us with a preoperative tracheostomy and had been excluded from the study as tracheostomy is known to affect swallowing function by interfering with laryngeal elevation. Also, during preoperative counseling, the parents of two kids refused the definitive surgery at this young age and we performed a tracheostomy. We did not involve these two kids also in our work.

### Surgery

Double-stage laryngotracheal reconstruction (LTR) was performed in all kids with technical modifications according to the case. Rib grafts were used in all cases older than 6 months of age and whenever posterior grafts were indicated (visual impression of small interarytenoid distance). In younger kids with only anterior grafts, alar cartilage was the selected grafting source. Submucosal cricoid arch trimming was done in one case of grade 4 webs. We always used refashioned Foley catheters as a suprastomal stent as routinely done in our practice [7]. We strived not to do full laryngofissure to any of our cases to maximize voice outcome. After the cricoid split, the soft-tissue component of the web was cut strictly in the midline from below under vision using a knife preserving the future anterior commissure. The stent was left in place for 6 weeks, and then, the cavity was endoscopically treated till decannulation.

### Swallowing evaluation

Evaluation of the swallowing function was done by clinical swallowing evaluation including symptoms and signs of swallowing dysfunction during feeding, such as vomiting, coughing, choking, or cyanosis, and bedside swallowing assessment using the 3-oz water swallow test [8]. Instrumental evaluation of swallowing function was performed using flexible endoscopic evaluation of swallowing (FEES). Assessment of the swallowing function using FEES was done based on the Cincinnati Children’s Hospital Medical Center (CCHMC) FEES protocol in infants and children [9]. Since all of our children were below 2 years, we used only liquid consistencies. The pooling of secretions was recorded and the Penetration-Aspiration scale was used to score the depth of laryngeal penetration and aspiration [10]. The accumulation of saliva and pharyngeal residue postswallow was also registered and we used the Yale Pharyngeal Residue Scale to rate the severity of pharyngeal residue [11]. The evaluation was performed both preoperatively and postoperatively after the removal of the stent and decannulation of patients.

### Voice evaluation

The severity of dysphonia was evaluated by auditory perceptual assessment of the children’s cry using the modified GRBAS scale [12]. The severity of dysphonia was scored from 0 to 3, where 0 was considered normal voice, while 3 represented severe dysphonia.

### Statistical analysis

Data entry and data analysis were done using SPSS version 24 (Statistical Package for Social Science). Data were presented as numbers, percentages, mean, and standard deviations. Chi-square test was used to compare qualitative variables between groups. The *P* value was considered statistically significant when *P* < 0.05.

## Results

Our study included 12 children, 7 (58.33%) were females. Half of our cases had comorbidities, where chromosome 22q11.2 microdeletion syndrome was the most common (Table 1).

**Table 1:** Demographic and clinical characteristics of the studied group

Patients characteristics		Study group (*n* = 12)
Age/months (mean ± SD)		11.92 ± 4.35
Weight in kg (mean ± SD)		8.67 ± 1.83
Sex		
Male		5 (41.67%)
Female		7 (58.33%)
Comorbidity		
Yes		6 (50%)
22q11	3 (25%)	
VSD	1 (8.33%)	
Orofaciodigital syndrome	1 (8.33%)	
ASD	1 (8.33%)	
No		6 (50%)
Type Cohen		
Type 3	5 (41.67%)	
Type 4	7 (58.33%)	

*SD* standard deviation, *VSD* ventricular septal defect, *22q11* chromosome 22q11.2 microdeletion syndrome, *ASD* ventricular septal defect

Four (33.33%) out of 12 children did not suffer from any symptoms or signs of swallowing difficulties on clinical evaluation of swallowing preoperatively versus postoperatively and their FEES examinations were normal as well. While eight (66.67%) children exhibited manifestations of swallowing difficulties on clinical swallowing evaluation and demonstrated evidence of swallowing dysfunction on their FEES.

On comparing the results of clinical swallowing evaluation pre- and postoperatively, the number of children suffering from swallowing difficulties had significantly increased during the postoperative evaluation where 6 (50%) children demonstrated choking during feeding after surgical division of the web in comparison to only 3 (25%) preoperatively. Also coughing and choking during the 3-oz water swallow test significantly increased following the division of the web with *P* < 0.001. Other data on clinical swallowing evaluation are shown in Table 2.

**Table 2:** Clinical swallowing evaluation of the studied subjects

Parameter	Preoperative (*n* = 12)	Postoperative (*n* = 12)	*P* value
1. Normal	4 (33.33%)	4 (33.33%)	*P* = 1
2. Vomiting	1 (8.33%)	4 (33.33%)	*P* < 0.001**
3. Coughing	3 (25.0%)	6 (50.0%)	*P* < 0.02*
4. Choking	3 (25.0%)	6(50.0%)	*P* < 0.02*
5. Cyanosis/congestion during feeding	4 (33.33%)	0	*P* < 0.000***
6. Diminished interest in feeding	1 (8.33%)	4 (33.33%)	*P* < 0.000***
7. Coughing/choking during the 3-oz water swallow test	2 (16.67%)	8 (66.66%)	*P* < 0.000***
8. Cyanosis during the 3-oz water swallow test	1 (8.33%)	0	*P* = 0.776

Flexible endoscopic examination of swallowing (FEES) of children with high grade glottis web revealed that the most prominent swallowing pathology was diminished laryngeal adductor reflex (LAR) which was recorded in 5 (41.67%) children. Swallow onset time was delayed to 3 s in 2 (16.67%) children, consistent penetration with liquids was observed in 2 (16.67%) which was contacting the aryepiglottic fold in 1 child and apparent through the interarytenoid notch in the other. A minimal amount of aspiration with liquids occurred following swallowing in 2 (16.67%) children, passing to the level of the vocal fold in 1 (8.33%) and contacting the aryepiglottic fold in the other child (8.33%). Silent aspiration was recorded in 1 (8.33%) child, and 3 (25.99%) children experienced pharyngeal residue following swallow. Table 3 shows the detailed swallowing pathologies in the preoperative FEES examination.

**Table 3:** Preoperative swallowing pathologies (*N* = 12)

Swallowing parameter	Yes (%)	No (%)
1. Laryngopharyngeal sensation		
Diminished laryngeal adductor reflex	5 (41.67%)	7 (58.33%)
Absent pharyngeal adductor reflex	2 (16.67%)	10 (83.33%)
2. Swallowing onset time		
Delay onset of swallow with liquids	2 (16.67%)	10 (83.33%)
Bolus head in vallecular space with liquids	2 (16.67%)	10 (83.33%)
Bolus head in the pyriform region with liquids	4 (33.33%)	8 (66.67%)
Combined vallecular and pyriform region	2 (16.67%)	10 (83.33%)
3. Laryngeal penetration		
Inconsistent	1(8.33%)	11 (91.67%)
Consistent	2 (16.67%)	10 (83.33%)
Contact with the aryepiglottic fold	1 (8.33%)	11 (91.67%)
Interarytenoid notch	1 (8.33%)	11 (91.67%)
Consistent protective responses	10 (83.33%)	2 (16.67%)
4. Aspiration		
Following swallow	2 (16.67%)	10 (83.33%)
Contact with aryepiglottic fold	1 (8.33%)	11 (91.67%)
Level of the true vocal fold	1 (8.33%)	11 (91.67%)
Minimal amount of aspiration	2 (16.67%)	10 (83.33%)
Consistent protective responses	12 (100%)	0
5. Silent aspiration	1 (8.33%)	11(91.67%)
6. Pharyngeal residue		
Vallecular residue with liquids	2(16.67%)	10 (83.33%)
Pyriform residue with liquids	1 (8.33%)	11 (91.67%)
Required multiple swallows to clear	0	12 (100%)

Postoperative evaluation of swallowing function demonstrated the following pathologies; diminished laryngopharyngeal sensation with impaired LAR in 8 (66.67%) and lost in 4 (33.33%) children. Also, swallow onset time with liquids was delayed to 4 s in 8 (66.67%) children. Consistent penetration with liquids was noticed in 8 (66.67%) children and it was observed contacting the aryepiglottic fold in approximately half of them. Postdeglutitive aspiration of a minimal amount of liquid was noticed in 5 (41.67%) children. Nevertheless, a moderate amount of aspiration with liquids was also recorded in 2 (16.67%) children. Five (41.67%) children showed silent aspiration for liquids. Other postoperative swallowing pathologies are given in Table 4.

**Table 4:** Postoperative swallowing pathologies (*N* = 12)

Swallowing parameter	Yes (%)	No (%)
1. Laryngopharyngeal sensation		
Diminished laryngeal adductor reflex	8 (66.67%)	4 (33.33%)
Absent pharyngeal adductor reflex	4 (33.33%)	8 (66.67%)
2. Swallowing onset time		
Delay onset of swallow with liquids	8 (16.67%)	4 (33.33%)
Bolus head in vallecular space with liquids	5(16.67%)	7 (58.33%)
Bolus head in the pyriform region with liquids	8 (33.33%)	4(33.33%)
Combined vallecular and pyriform region with liquids	5 (16.67%)	7 (58.33%)
3. Laryngeal penetration with liquids		
Inconsistent	2 (16.67%)	10 (83.33%)
Consistent	8 (66.67%)	4 (33.33%)
Contact with the aryepiglottic fold	4 (33.33%)	8 (66.67%)
Interarytenoid notch	3(25.00%)	9 (75.00%)
Level of the true vocal fold	1 (8.33%)	11 (91.67%)
Consistent protective responses	4 (33.33%)	8 (66.67%)
4. Aspiration		
Following swallow	5 (41.67%)	7 (58.33%)
Contact with aryepiglottic fold	3 (25.00%)	9 (75.00%)
Level of the true vocal fold	2 (16.67%)	10 (83.33%)
Minimal amount of aspiration	3 (25.00%)	9 (75.00%)
Moderate amount of aspiration	2 (16.67%)	10 (83.33%)
Consistent protective responses	4 (33.33%)	8 (66.67%)
5. Silent aspiration	5 (41.67%)	7 (58.33%)
6. Pharyngeal residue		
Vallecular residue with liquids	3 (16.67%)	9 (75.00%)
Pyriform residue with liquids	2 (8.33%)	10 (83.33%)
Required multiple swallow to clear	1 (16.67%)	11 (91.67%)

A deterioration in the swallowing function had occurred in children who exhibited swallowing pathologies in their preoperative FEES, especially in the scores of the penetration-aspiration scale which had significantly increased with *P* < 0.000 after surgical intervention. Whereas, the severity of the pharyngeal residue postswallow had not much altered after the intervention. Tables 5, 6, and 7 demonstrate the impact of surgical division of the web on the swallowing function as regards the penetration-aspiration scores and pharyngeal residue postswallow ratings.

**Table 5:** Impact of surgical division of the web on penetration-aspiration scores of the study subjects

Swallowing parameter	Preoperative *N* = 12	Postoperative N = 12	*P* value
1. Penetration-aspiration			
PAS 1	1(8.33%)	0(8.33%)	
PAS 2	2 (16.67%)	4 (25.00%)	*P* < 0.001***
PAS 3	1 (16.67%)	2 (16.67%)	
PAS 4	0	1 (8.33%)	
PAS 5	0	0	
PAS 6	0	0	
PAS 7	0	0	
PAS 8	0	5(41.66%)	
Total	0.66 ± 0.14	4.833 ± 1.38	*P* < 0.001

**Table 6:** Impact of surgical division of the web on the severity of vallecular residue

Grade	Preoperative *N* = 12	Postoperative *N* = 12	*P* value
I (none)	10 (83.33%)	9 (75.00%)	
II (trace)	1	2 (16.67%)	*P* = 0.248 (NS)
III (mild)	1	1 (8.33%)	
IV (moderate)	0	0	
V (severe)	0	0

**Table 7:** Impact of surgical division of the web on the severity of pyriform residue

Grade	Preoperative *N* = 12	Postoperative *N* = 12	*P* value
I (none)	11 (91.67%)	10 (83.33%)	*P* = 0.582 (NS)
II (trace)	1 (8.33%)	1 (8.33%)	
III (mild)	0	1 (8.33%)	
IV (moderate)	0	0	
V (severe)	0	0	

As regards the severity of dysphonia, 1 (8.33%) case had grade 2, 7 (58.34%) cases had grade 3 dysphonia, and 4 (33.33%) cases were completely aphonic on their preoperative voice evaluation. The severity of dysphonia had significantly improved on the postoperative evaluation where 1 (8.33%) case showed normal voice, 8 (66.66%) cases had grade 1, and only 3 (25%) cases had grade 2 dysphonia**. **Table 8 shows the effect of surgical division of the web on the severity of dysphonia.

**Table 8:** Impact of surgical division of the web on the severity of dysphonia

Dysphonia grade	Preoperative *N* = 12	Postoperative *N* = 12	
0	0	1 (8.33%)	*P* = 0.001**
1	0	8 (66.66%)	
2	1 (8.33%)	3 (25%)	
3	7 (58.34%)	0	
Aphonia	4 (33.33%)	0	

0 = normal voice, 1 = mild dysphonia, 2 = moderate dysphonia, 3 = severe dysphonia

Concerning decannulation, serial endoscopies were done until proper healing and disappearance of occluding granulations were observed followed by decannulation. We needed to do balloon dilatation for three of our kids before achieving decannulation. All the kids (100%) were decannulated successfully.

## Discussion

The upper aerodigestive tract (UADT) evolves in tandem with the underlying framework [13]. The process of swallowing requires a high level of complex coordinated reflex-mediated neuromuscular factors to efficiently control food and fluid and prevent it from passing to the airway [14]. Children with congenital airway anomalies might encounter swallowing difficulties due to the lack of pulmonary reserve, thus impeding the coordinated sucking, swallowing, and breathing which compromises airway protection mechanisms. Many cases with high-grade glottic webs give a history of recurrent hospital admissions not explainable by the degree of upper airway compromise. When reviewing their medical records, we found that aspiration pneumonia was a frequent pathology, so swallowing assessment was a must.

Our study included 12 children with age 11.92 ± 4.35 months. Half of our cases had comorbidities, where chromosome 22q11.2 microdeletion syndrome was the most common. Lawlor et al. [15] had 3.7 years as an average age at diagnosis and underlying anomalies included congenital heart diseases, subglottic stenosis, 22q11.2 deletion syndrome, and recurrent respiratory papillomatosis. Cheng et al. [16] showed confirmatory genetic evidence of deletion of chromosome 22q11.2 in more than 60% of their cases.

De Trey et al. [6] had comorbidities in eight patients (57%); the 22q11.2 microdeletion syndrome was discovered in 29% of the cases. Aortic valve dysplasia, ventricular septal defect, and orofaciodigital syndrome type 8 were also documented.

Fokstuen [17] was the first to recognize the glottic web as a symptom of chromosome 22q11.2 microdeletion syndrome. Cardiovascular diseases, such as aortic arch abnormalities and vascular rings [18], are also known to be related with glottic webs and were detected in their patient sample. As a result, it is critical to look for diseases associated with the glottic web.

There are two distinct classification systems to stratify the glottic web. Benjamin developed a staging system according to the location of the web into glottic, supraglottic, subglottic, and interarytenoid stenoses [19].On the other side, Cohen’s staging system depends on the degree of stenosis, Cohen’s type 1 glottis involves less than 35% of the anterior web, type 2 involves 35–50%, type 3 (50–75%), and type 4 involves 75–90% [4]. We included higher grades of the glottic web; grades 3 and 4 according to Cohen’s classification.

The severity of dysphonia significantly improved in the postoperative evaluation in this study. Voice evaluation in the pediatric population is difficult and several pieces of research have been conducted to investigate the quality of voice after LTR [20]. The severity of dysphonia was evaluated in this study by auditory perceptual assessment of the children’s cries using the modified GRBAS scale [12]. De Trey et al. [6] showed improvement in the voice after LTR.

LTR changes the morphology of the larynx and may affect laryngeal closure, necessitating compensation of the existing structures for airway protection. The degree of aspiration is related to the completeness of laryngeal closure and overall swallowing coordination [21]. Willging et al. discovered that those who had further surgical procedures involving supraglottic tissues had the greatest duration of swallowing problems [22]. Additionally, we postulate that with glottic and subglottic expansion, there is added impedance of the glottic closure reflex which together with the lack of coordination between suck, breathing, and swallowing have led to the exaggeration of the swallowing dysfunction. According to some studies, despite significant surgical modification of the laryngotracheal complex, long-term dysphagia is uncommon [23, 24].

In this study, we evaluated swallowing function in infants undergoing surgical division of high grade glottic web where children with normal swallowing preoperatively did not manifest any swallowing difficulties postoperatively/after stent removal. On the other hand, swallowing symptoms were worsened after stent removal in infants who exhibited abnormalities in swallowing function as compared to their preoperative evaluation. Five cases encountered silent aspiration and were considered unsafe swallow, for them a nasogastric tube was inserted, while for the remaining cases, dietary modifications and compensatory rehabilitative strategies were advised. Six months postoperatively, all kids were considered safe swallow and were able to feed orally. De Trey et al. [6] showed that swallowing was normal in all their cases except one patient who still was percutaneous endoscopic gastrostomy dependent at the last follow-up as was the case preoperatively. None of the other cases had broncho-aspiration after surgery.

The primary predictor of postoperative feeding status is considered to be preoperative feeding status [22, 25]. Independent of feeding challenges, risk factors include being younger than 2 years old, having a tracheostomy, and having many medical comorbidities [13, 22, 25].

The aggravating swallowing dysfunction following stent removal is probably due to loss of the proprioception with muffling of the glottic closure reflex due to the presence of the tracheostomy. We also believe that tracheostomy has disrupted the phasic glottic function necessary for the initiation of the glottic closure reflex. Moreover, an existing tracheostomy impedes the elevation of the subglottic pressure which has an inhibitory effect on the respiratory muscles, hence, predisposing them to penetration and aspiration during swallowing [26].

Preoperative swallowing exams are critical for identifying at-risk patients as well as those who have pre-existing swallowing difficulties that may be aggravated by surgery. It aids in modifying postoperative assessment procedures, creating and implementing feeding programs, allowing for early oral feeding, and the preoperative swallowing exam is a predictor of prolonged hospitalization in 5% of patients [21, 23, 25]. Although transitory dysphagia is frequent after LTR, no studies have found a link between preoperative feeding abilities and postoperative airway protection capacities [13].

Abnormalities with oral feeding usually resolve with the regaining of airway patency; nevertheless, postoperative swallowing evaluation is necessary to ensure safe swallowing, especially during the transition phase to oral swallowing and complete decannulation.

Clinical evaluation is useful both before and after surgery to identify the signs of aspiration, assess oral feeding, identify behavioral components, the ability to deal with oral secretions with spontaneous swallows, and difficulty with swallowing [13, 21, 22]. Prolonged aspiration, even if mild, can cause recurrent cough, bronchitis, and pneumonia [27, 28]. All subjective clinical signs, on the other hand, have been demonstrated to have high sensitivity with poor specificity and a positive predictive value of roughly 80% [29]. The reduced pharyngeal and laryngotracheal feeling can cause silent aspiration, which is undetectable in the clinical swallowing evaluation [14].

We used the fiberoptic endoscopic evaluation of swallowing (FEES) as instrumental tests, such as FEES, provide particular information about both the sensory and motor aspects of swallowing, as well as airway closure integrity and timing during swallowing. It has been shown to have similar or higher sensitivity than the videofluoroscopic swallow study in identifying both laryngeal penetration and tracheal aspiration [29].

To our best knowledge, it is the first prospective study to evaluate the swallowing function in children with higher grades of glottic web and to detect the impact of surgical division of the glottic web on the swallowing parameters. The limitation of our work is the small sample due to the rarity of the disease.

## Conclusion

Swallowing assessment is mandatory as children with higher grades of the glottic web, requiring reconstructive surgeries, are at risk of swallowing deficit which can be aggravated postoperatively. Swallowing deficit is not a contraindication for surgery, but implementing preoperative feeding programs and proper counseling of parents should be performed. With improvement in the airway and surgery-specific outcomes, swallowing function is an important secondary outcome that has a significant impact on the lives of these kids and their families.

## Data Availability

The data sets used and/or analyzed during the current study are available from the corresponding author upon reasonable request.
